# Supplementary material for: Causal Relationship between Aging and Anorexia Nervosa: A White-Matter-Microstructure-Mediated Mendelian Randomization Analysis
Source: Biomedicines. 2024 Aug 16;12(8):1874. doi: 10.3390/biomedicines12081874 (PMC11351342; doi:10.3390/biomedicines12081874)
Supplement: Supplementary file 1 [file biomedicines-12-01874-s001.zip › Supplementary Figure/Figure S4/Singe SNP plot of MR analysis for TL to ubm-b-1996.pdf]

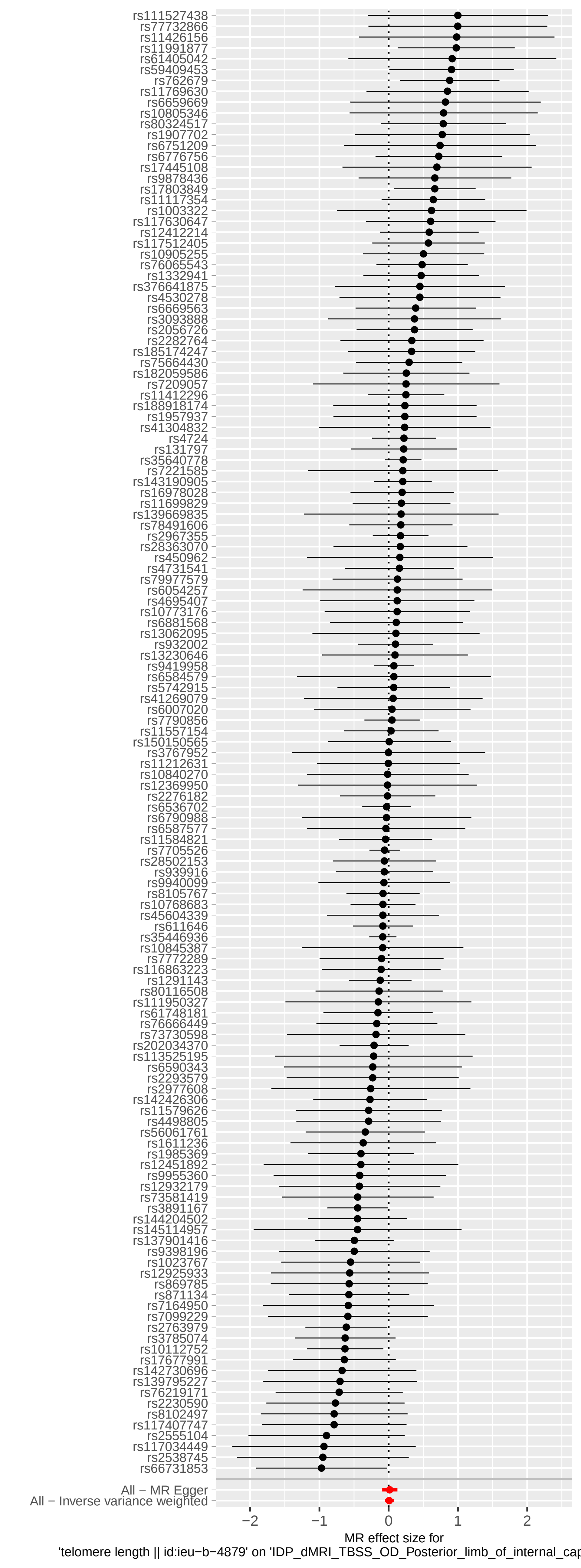

rs111527438
rs77732866
rs11426156
rs11991877
rs61405042
rs59409453
rs762679
rs11769630
rs6659669
rs10805346
rs80324517
rs1907702
rs6751209
rs6776756
rs17445108
rs9878436
rs17803849
rs11117354
rs1003322
rs117630647
rs12412214
rs117512405
rs10905255
rs76065543
rs1332941
rs376641875
rs4530278
rs6669563
rs3093888
rs2056726
rs2282764
rs185174247
rs75664430
rs182059586
rs7209057
rs11412296
rs188918174
rs1957937
rs41304832
rs4724
rs131797
rs35640778
rs7221585
rs143190905
rs16978028
rs11699829
rs139669835
rs78491606
rs2967355
rs28363070
rs450962
rs4731541
rs79977579
rs6054257
rs4695407
rs10773176
rs6881568
rs13062095
rs932002
rs13230646
rs9419958
rs6584579
rs5742915
rs41269079
rs6007020
rs7790856
rs11557154
rs150150565
rs3767952
rs11212631
rs10840270
rs12369950
rs2276182
rs6536702
rs6790988
rs6587577
rs11584821
rs7705526
rs28502153
rs939916
rs9940099
rs8105767
rs10768683
rs45604339
rs611646
rs35446936
rs10845387
rs7772289
rs116863223
rs1291143
rs80116508
rs111950327
rs61748181
rs76666449
rs73730598
rs202034370
rs113525195
rs6590343
rs2293579
rs2977608
rs142426306
rs11579626
rs4498805
rs56061761
rs1611236
rs1985369
rs12451892
rs9955360
rs12932179
rs73581419
rs3891167
rs144204502
rs145114957
rs137901416
rs9398196
rs1023767
rs12925933
rs869785
rs871134
rs7164950
rs7099229
rs2763979
rs3785074
rs10112752
rs17677991
rs142730696
rs139795227
rs76219171
rs2230590
rs8102497
rs117407747
rs2555104
rs117034449
rs2538745
rs66731853
All − MR Egger
All − Inverse variance weighted
−2
−1
0
1
2
MR effect size for
'telomere length || id:ieu−b−4879' on 'IDP_dMRI_TBSS_OD_Posterior_limb_of_internal_cap
